# Septic AKI in ICU patients. diagnosis, pathophysiology, and treatment type, dosing, and timing: a comprehensive review of recent and future developments

**DOI:** 10.1186/2110-5820-1-32

**Published:** 2011-08-09

**Authors:** Patrick M Honore, Rita Jacobs, Olivier Joannes-Boyau, Jouke De Regt, Willem Boer, Elisabeth De Waele, Vincent Collin, Herbert D Spapen

**Affiliations:** 1 Intensive Care Department, Universitair Ziekenhuis Brussel, Vrije Universitieit Brussel (VUB), 101, Laarbeeklaan, 1090 Jette, Brussels, Belgium; 2 Departement d'Anesthesie-Reanimation II (DAR II), Haut Leveque University Hospital of Bordeaux, University of Bordeaux 2, Pessac, France; 3 Department of Anaesthesiology and Critical Care Medicine, Ziekenhuis Oost-Limburg, Genk, Belgium; 4 Intensive Care Unit, Cliniques de l'Europe-Site St Michel, Brussels, Belgium

**Keywords:** Hemofiltration, Acute Kidney Injury, Pathphysiology, Dosing, Timing, Diagnosis, Review, CRRT, Dialysis, Septic Acute Kidney Injury, Sepsis, SIRS

## Abstract

Evidence is accumulating showing that septic acute kidney injury (AKI) is different from non-septic AKI. Specifically, a large body of research points to apoptotic processes underlying septic AKI. Unravelling the complex and intertwined apoptotic and immuno-inflammatory pathways at the cellular level will undoubtedly create new and exciting perspectives for the future development (e.g., caspase inhibition) or refinement (specific vasopressor use) of therapeutic strategies. Shock complicating sepsis may cause more AKI but also will render treatment of this condition in an hemodynamically unstable patient more difficult. Expert opinion, along with the aggregated results of two recent large randomized trials, favors continuous renal replacement therapy (CRRT) as preferential treatment for septic AKI (hemodynamically unstable). It is suggested that this approach might decrease the need for subsequent chronic dialysis. Large-scale introduction of citrate as an anticoagulant most likely will change CRRT management in intensive care units (ICU), because it not only significantly increases filter lifespan but also better preserves filter porosity. A possible role of citrate in reducing mortality and morbidity, mainly in surgical ICU patients, remains to be proven. Also, citrate administration in the predilution mode appears to be safe and exempt of relevant side effects, yet still requires rigorous monitoring. Current consensus exists about using a CRRT dose of 25 ml/kg/h in non-septic AKI. However, because patients should not be undertreated, this implies that doses as high as 30 to 35 ml/kg/h must be prescribed to account for eventual treatment interruptions. Awaiting results from large, ongoing trials, 35 ml/kg/h should remain the standard dose in septic AKI, particularly when shock is present. To date, exact timing of CRRT is not well defined. A widely accepted composite definition of timing is needed before an appropriate study challenging this major issue can be launched.

## Introduction

Continuous renal replacement therapy (CRRT) and especially continuous veno-venous hemofiltration (CVVH) are widely used in intensive care units (ICU). However, broad consensus or distinct recommendations with regard to optimal use of CRRT in general, and particularly in non-septic acute kidney injury (AKI) beyond shock, are lacking. Nonetheless, interest in AKI and blood purification techniques in this field continues to grow as illustrated by many small studies and by an increasing number of multicenter, randomized controlled trials (RCTs) [1]. Regardless of ongoing clinical controversy [2,3], large epidemiological studies convincingly show that CVVH and derived techniques represent treatments of choice for the management of septic shock complicated by AKI [4]. Such epidemiological evidence is relevant because RCTs are no longer considered to be the absolute "gold standard" for demonstrating best evidence in a complex ICU setting [5].

This review was designed to address the most recent knowledge and future developments with regard to continuous and intermittent renal replacement therapy (IRRT) that are of practical interest to ICU physicians.

### New insights in the pathophysiology of septic AKI

Ample proof exists to sustain a more prominent role of apoptosis rather than pure necrosis in the pathophysiology of sepsis and septic shock [6]. Despite substantial advances in elucidating the etiology of tubular apoptotic lesions [7], studies that look for a possible key role of apoptosis in the mechanisms of organ dysfunction in humans have yielded conflicting results [8,9].

Apoptosis has been put forward as a major player in septic AKI [10]. However, histopathological studies are scarce and mostly date from before 1980 [9,11]. Kidney biopsies from 19 consecutive patients who died from septic shock were compared with post-mortem biopsies taken from 8 trauma patients and 9 patients with non-septic AKI [9].

Acute tubular apoptosis was demonstrated in septic AKI, whereas almost no apoptosis was detected in the non-septic AKI patients. In this study, three different techniques to confirm apoptosis were applied: routine microscopy, the TUNEL (Terminal-deoxynucleotidyltransferase-mediated d UTP-digoxigenin nick and labeling) method, and activated caspase 3 labeling. The two last techniques enabled to detect 6% apoptosis in the septic group versus only 1% in the non-septic group (*p *< 0.0001). However, some control biopsies were retrieved from patients who died at the scene of trauma, which may have limited the development of any type of histologic kidney lesion. On the other hand, a subset of six other controls consisted of out-of-hospital cardiac arrest patients who most likely had cardiogenic shock before dying. Therefore, the latter group may more reliably represent non-septic-AKI, including its accompanying hemodynamically related lesions. Further drawbacks of the study are the cadaveric nature of the biopsies, which precludes interpretation of potentially reversible changes that occur in recovering patients and some control groups being historical controls.

From a theoretical point of view, necrosis results from the additive effect of a number of independent biochemical events that are activated by severe depletion of cell energy stores. In contrast, the process of apoptosis follows a coordinated, predictable, and predetermined pathway. These biochemical differences between apoptosis and necrosis have important therapeutic implications. Once a cell has been severely injured, necrosis is difficult to prevent whilst the apoptotic pathway can be modulated to maintain cell viability [12]. Theoretically, the components of the apoptotic pathway that could be amenable to therapeutic modulation are numerous [13,14]. When considering septic AKI as a disease entity in which hemodynamic compromise of the kidney alone is no longer the rule, a revision of the "prerenal" AKI concept must be anticipated. Several assumptions associated with the "prerenal azotemia paradigm" will be in violation of this revised definition [15]. It is not evidenced that acute tubular necrosis (ATN) is the histopathological substrate of septic AKI and, above all, it is not proven that urine tests can discriminate between functional and structural AKI.

### Diagnosis of AKI and potential role of biomarkers

Early recognition of AKI in the ICU setting is crucial. Indeed, AKI has become a major issue with rising incidence causing more than four million deaths per year worldwide [16]. Also, the lack of an early and reliable biomarker for AKI causes significant delay in initiating appropriate therapy [16]. This is in contrast with the "biologic revolution" in cardiology, which produced various markers (including troponin) for early diagnosis of cardiac damage that enabled early and effective treatment [17]. In fact, standard evaluation of renal function still largely depends upon serum creatinine and serum creatinine-based formulas, which were primarily designed for longitudinal assessment of baseline renal function [18-20]. An accurate diagnosis of AKI is even more problematic in critically ill patients. The instability of renal function in this population significantly reduces the validity of measures based on creatinine assessment [21,22]. Until recently, studies of AKI used various creatinine-based equations designed to evaluate baseline renal function [23]. This approach is not adequate in the critically ill. A more accurate and sensitive description of AKI and a multidimensional AKI classification system are definitely needed. Basically, such system should grade AKI severity.

At present, the most widely used classification is the RIFLE model (acronym for Risk, Injury, Failure, Loss, End-stage renal disease) [24], proposed by the Acute Dialysis Quality Initiative group. Hoste et al. [25] validated the RIFLE criteria in critically ill adults, showing that patients with maximum RIFLE class R, I, and F had hospital mortality rates of respectively 8.8%, 11.4%, and 26.3% [25]. Finally, recognition of AKI before changes in serum creatinine occur has been and remains an area of current intensive research. The rationale comes from studies demonstrating that even small or relative increases in serum creatinine are associated with increased patient morbidity and mortality, independently from expected calculated mortality.

As anticipated, Chertow et al. recently found a nearly sevenfold increase in the odds of death in patients with a 0.5 mg/dL increase in serum creatinine, even when adjusted for numerous comorbidities [26]. The search for valuable and early markers for AKI in critically ill patients must be prioritized because critically ill patients are dying from and not just "with" AKI, underscoring that AKI is an independent risk factor for mortality; indeed, acute kidney injury network (AKIN) was conceived to better tackle this important issue [24,25]. To date, however, the development of an early marker for renal dysfunction in the ICU remains elusive [17]. Serum creatinine concentrations and creatinine clearance are unreliable indicators for acute and abrupt changes in kidney function [27]. Serum creatinine only comes into play as a marker for decreasing kidney function after more than 50% of kidney function has been lost. It is only useful after a steady state has been reached, which can take considerable time in ICU patients [28]. Creatinine clearance somewhat better reflects changes in kidney function. However, loss of kidney function occurs at a late phase in the process of cellular kidney damage, which explains why a decrease in creatinine clearance detects AKI with a delay of many hours [29].

Human studies have shown that AKI can be prevented and treated if adequate measures are undertaken shortly after the initial kidney insult [30]. As such, they stress the need to obtain an early biomarker of kidney injury in the ICU for optimization of timely treatment [31].

Biomarkers also may become tools to better differentiate AKI etiology, thus directing more appropriate treatment [32]. An early biomarker might have prognostic implications, given the immense impact of AKI-associated mortality on global disease-related mortality [33,34]. Unfortunately, all actually studied biomarkers have insufficient sensitivity to detect AKI in the ICU, and diagnostic power may only increase when a combination of various biomarkers is used, including cystatin-C, Neutrophil gelatinase-associated lipocalin (NGAL) in the serum and urine, IL-18, and kidney injury molecule 1 (KIM -1) [34-36].

### Type of therapy in a general ICU setting

CRRT in ICU patients can be provided as continuous veno-venous hemodiafiltration (CVVHDF), CVVH, or in a hybrid form, such as high-volume CVVH. IRRT options include sustained low-efficiency dialysis (SLED), intermittent hemodialysis (IHD), or others. Much debate is ongoing about choice of epuration mode (continuous or intermittent), choice of technique (diffusion or convection), or eventual combination of techniques (diffusion on top of convection) [37]. In fact, CVVH, CVVHDF, and related techniques are more suitable for indications, such as hemodynamic instability, hepatorenal syndrome, raised intracranial pressure, and (sub)acute or extreme fluid overload. Likewise, intermittent therapies have proper advantages [38,39]. However, many intensivists are inclined to employ continuous methods, especially in hemodynamically unstable patients. This attitude gained more acceptance after publication of a meta-analysis, which showed less hemodynamic instability and better control of fluid balance with CRRT [40,41]. Furthermore, a recent study of the PICARD group showed that CRRT rather than any intermittent or semi-continuous method was able to remove extra fluid in severely fluid-overloaded AKI patients [42]. Although some groups have reported no overt problems with IHD in unstable ICU patients [42-44], most opinion leaders consider CRRT as the most appropriate therapy in vasopressor-dependant patients with AKI. This recommendation is supported by aggregated data of the ATN and RENAL studies, indicating that vasopressor-dependant ICU patients on CRRT will less frequently evolve to chronic dialysis than patients who receive intermittent therapies [45]. The same opinion leaders also recommend CRRT during the acute phase of AKI, particularly in patients with severe hemodynamic instability or when extensive fluid removal may allow more effective drug therapy [40,42,46]. Admittedly, two recent RCTs did not show superiority of CRRT to IHD [2,3]. Indeed, the Hemodiafe study [2], comparing IHD with CVVHDF in ICU patients, showed that both techniques were comparable in terms of patients' outcome. This was confirmed by a subsequent RCT [3]. Only a few, small, randomized and observational studies did not find a difference between SLED and CRRT [47,48]. A recent paper that reviewed the data discussed during the preparation of the most recent KDIGO guidelines gave CRRT and SLED the same level of evidence [49], although there are only very few randomized studies comparing SLED and CRRT, whereas there are large numbers of randomized studies that compare IHD and CRRT. In other words, the level of evidence is much weaker for SLED. As a consequence, many experts in the field still recommend CRRT and not SLED in hemodynamically unstable patients [37].

### Choosing between diffusion and convection--still an ongoing dilemma but with light at the end of the tunnel

Diffusion permits removal of small molecules and an excellent ionic equilibration, whereas convection more efficiently eliminates middle-large molecules. However, when a high cut-off membrane is employed, dialysis techniques also can remove larger molecules, which renders the difference between convection and diffusion very artificial [50-52]. To date, no superiority of one method over the other has been demonstrated in terms of improved outcome, except for some small studies that have reported better performance of large pore membranes [53,54].

Also, classic membranes have not been compared on a large scale with the newest large pore membranes (e.g., Septe*X*^®^; Gambro™) [55]. A new generation of membranes has emerged that specifically focuses on endotoxin adsorption (Toraymyxin^®^, Toray™ and Oxiris^®^, Gambro™) or immunoadsorption (Prosorba^®^, Fresenius™). Recent studies using these membranes were promising [56], but larger RCTs are awaited. Additionally, the use of citrate [57] and a larger surface membrane [58] have both been shown to narrow the gap between diffusion and convection principles for removing middle-large molecules.

### Type of dose provision in septic and non-septic AKI

Two recent, large RCTs provided more insight in the dosing of fluid exchange. The pivotal study by Ronco et al. in ICU patients recommended a hemofiltration dose of 35 ml/kg/h [59].

This association between increasing renal replacement dose (RRT) and better outcome was further addressed by two, large, randomized, landmark studies. One study found better outcome in patients treated by hemodialysis on a daily base rather than three times per week [60]. Essentially, this study underscored that an IHD session of 4-h duration repeated three times per week was not an adequate treatment for ICU-acquired AKI. Approximating the more efficient dosing regimen found by Ronco, the other study showed that increasing convection dose and adding dialysis to hemofiltration improved outcome [61]. However, a recent RCT (acute renal failure trial network (ATN study)) conducted by Palevsky et al. showed that less intensive therapy (IHD three times per week, CVVHDF at 20 ml/kg/h, or SLED for unstable patients) was comparable to intensive therapy (daily hemodialysis or CVVHDF at 40 ml/kg/h for unstable patients) in terms of mortality at 90 days [62]. Nonetheless, this study had several limitations. More than 65% of the patients already received intermittent therapy before being allocated to a specific treatment group [63,64]. Intervention also was extremely delayed, which could have had a negative impact on outcome of the intensive therapy group [63,64]. Finally, the relatively low rate of continuous therapy compared with other large, randomized studies may have influenced the renal recovery rate [45]. A more convincing answer regarding the dose for non-septic AKI came from the so-called "randomized evaluation of normal versus augmented levels" (RENAL) study, which demonstrated no beneficial effect of CVVHDF at 40 ml/kg/h compared with 25 ml/kg/h [65]. Therefore, current consensus suggests a hemofiltration dose of 25 ml/kg/h in nonseptic AKI with no additional benefit from a dose increase. However, some important issues must be stressed. First, expert opinion imposes that patients should not be undertreated and should receive at least 25 ml/kg/h of fluid exchange. In practice, this implies prescribing 30-35 ml/kg/h to account for predictable (bags change, nursing...) or unpredictable (surgery, clotting...) breaks in treatment [4,66]. Second, the amount of dose to be used in septic AKI remains questionable; some small, prospective, randomized studies have shown a beneficial effect of high-dose hemofiltration. The multicenter "IVOIRE study" (hIgh Volume in Intensive care), which compared hemofiltration doses of 35 ml/kg/h versus 70 ml/kg/h in patients with sepsis-induced shock, AKI, and multiple organ failure, may settle this debate in the near future. Although patients included were more severely ill, overall mortality in the IVOIRE study remains very low (39% at 28 days and 52% at 90 days) compared with the RENAL study. This may be due to the earlier start of treatment at the renal injury level [67]. Awaiting results from this important trial, 35 ml/kg/h should remain the standard dose in septic AKI, particularly in the presence of shock.

### The complex issue of "timing of CRRT" during AKI in ICU patients

The right time to start RRT is still a topic of debate. The main reasons are the absence of a clear and consensual definition of AKI to stratify patients according to the degree of renal impairment and the difficulty to obtain homogeneous patient groups for study purposes. The development of two new classifications, RIFLE and AKIN, represents a major step forward [68]. Both classifications alert clinicians of the presence of AKI and allow early intervention.

Several recent studies and meta-analyses fuelled interest for early start of RRT, but large RCTs about this topic are still awaited. One RCT regarding timing of hemofiltration (defined as the time between ICU admission and start of therapy) was negative [69]. However, this trial was insufficiently powered and included a very selective post-cardiac surgery population. Experts recommend beginning RRT earlier, particularly in sepsis where AKI is known to be rapidly progressive. However, a French RCT [70] showed the opposite and a Belgian study raised concern about a potentially harmful effect of starting RRT too early [71]. It seems acceptable to start RRT at the RIFLE injury stage (or AKIN stage 2) in septic AKI, especially when shock is present, but consensus on this topic awaits results from large-scale RCTs. Early use of RRT also may be relevant in patients, mainly pediatric, treated by extracorporeal membrane oxygenation for severe acute respiratory distress syndrome (ARDS) [72,73]. Fluid overload definitely plays a role in timing, because CRRT proved successful in patients without AKI but refractory to diuretics [72]. On top of this, new composite indices of timing have been proposed (e.g., severity of organ dysfunction (SOFA score), severity of AKI (RIFLE or AKIN stage), fluid overload status, time from admission, biomarker use, etc.), but their use in daily practice remains to be evaluated [74]. In that context, a recent, prospective study [75] and meta-analysis clearly favored early timing [76].

### Anticoagulation during CRRT: is citrate no longer the Achilles' heel?

Anticoagulation remains the topic of continuous development and research, in particular since the introduction of citrate and upcoming dedicated machines. Unfractionated heparin (UFH) remains the most commonly used anticoagulant for RRT worldwide, but citrate and other alternatives become increasingly important. Oudemans van Straaten et al. compared citrate to low molecular weight heparin (LMWH) anticoagulation in a predominantly surgical population [77]. Although no difference was found in terms of efficacy, an unexplained improved outcome was observed in the citrate-treated patients. In fact, 3-month mortality (63%) was unexpectedly high in the LMWH group, whereas outcome in the citrate group (48%) approached "usual" mortality. Antithrombin must be taken into consideration when using UFH during RRT, because it is a mandatory yet often neglected cofactor for coagulation. Antithrombin activity often is reduced necessitating supplementation in critically ill, particularly septic, patients [78]. A final issue concerns the replacement fluid. The composition of new products on the shelf more closely resembles that of plasma (e.g., by having phosphorus directly added to the fluid bag) [79], thereby avoiding the previously observed severe ionic disturbances [80].

### New perspectives for medical treatment of septic AKI

With septic AKI seen as an apoptotic-inflammatory AKI, the administration of caspase inhibitors (CI) gains increasing interest. Indeed, CI ameliorate ischemia-reperfusion injury in different organs, including the kidneys. However, it remains uncertain to what extent this protective effect of caspase inhibition is caused by reduced (intra-renal) inflammation or by less renal tubular cell loss due to apoptosis [7]. In addition to caspase inhibition, the apoptotic pathway offers many opportunities for therapeutic interventions that may prevent or reduce renal tubular cell apoptosis and, as such, renal function impairment. In a rat model of glycerol induced AKI (Gly-AKI) [81], caspases were found to participate in inflammation, apoptosis, vasoconstriction, and finally tubular necrosis. Early caspase inhibition attenuated these processes and significantly improved renal function [82]. Apoptosis also occurs in the kidney during LPS-induced AKI (LPS-I-AKI) [83], but its relative importance in this condition remains unproven. Guo and coworkers [83] hypothesized that treatment with a caspase inhibitor would protect mice from LPS-I-AKI. Mice, injected with LPS, were either treated with a broad-spectrum caspase inhibitor or placebo. Caspase inhibition was found to protect against LPS-I-AKI, not only by preventing apoptotic cell death but also by inhibiting inflammation [83]. The authors concluded that apoptotic kidney cells may act as a source of local inflammation producing subsequent non-apoptotic renal injury [83]. Another recent study [84] showed that plasma from septic burn patients with AKI could initiate pro-apoptotic effects and *in vitro *functional alterations of renal tubular cells and podocytes that correlated with the degree of proteinuria and renal dysfunction. In this model, additive and even synergistic effects of sepsis and burn injury were noticed. Further research for therapeutic interventions is warranted in several areas such as binding and elimination of endotoxin by extracting the source of sepsis, blocking of various apoptotic pathways, or even extracorporeal removal of circulating toxic mediators using high volume hemofiltration, high permeability hemofiltration and plasma filtration coupled with adsorption [85]. A recent study showed that extracorporeal therapy with polymyxin B therapy reduced the pro-apoptotic activity of plasma of septic patients on cultured renal cells, providing further evidence for a preponderant role of apoptosis in the development of sepsis-related AKI [86]. It seems likely that plasma separation techniques can prove beneficial in renal injury through the removal of pro-apoptotic factors and cytokines. In fact, the higher mortality of sepsis-associated AKI is not linked to intrinsic renal lesions alone but correlates well with remote sepsis-induced inflammatory tissue damage [87]. It is known that AKI, and sepsis-induced AKI in particular, may induce a state of immunoparalysis, thereby increasing the risk of subsequent infection [88]. Therefore, the inflammatory process accompanying septic AKI may substantially influence the dose of therapy [89] and, by removing pro-apoptotic factors, plasma separation could be positioned more as a preventive than as a treatment measure for AKI [88].

### New avenues in the "CRRT armamentarium" for septic AKI

At first glance, high permeability hemofiltration (HPHF) or even high-volume hemofiltration (HVHF) seem to be attractive alternatives to classical CRRT. Removal of higher molecular weight mediators is greatly enhanced by HPHF [90]. Since the early 1990s, it has been advocated that reducing cytokine levels in the blood compartment could theoretically lower mortality. Reducing the unbound cytokine load was logically assumed to limit remote organ damage, hence reducing overall mortality. However, recent insight in cytokine pharmacokinetics shows that this is an oversimplified generalization of a far more complex process, because it neglects the possible effects that changes in blood cytokines might have at the interstitial and tissue level [91]. In this setting, techniques promoting rapid and substantial removal of mediators are privileged. Such techniques include (very) HVHF as well as HPHF [92,93], super high flux hemofiltration (SHFHF), hemadsorption, and coupled plasma filtration and adsorption (CPFA) [94]. Because the clinical effectiveness of blood purification techniques could not be explained by a concomitant decline in blood mediator levels [95], interest shifted to the effects of HVHF at the interstitial and tissue level. Unfortunately, it is almost impossible to measure interstitial mediator levels and, as such, to quantify how HVHF interacts with the immune-inflammatory pathways to produce the observed significant dynamic changes in tissues and interstitium. Indirect evidence of a link between mediator level and subsequent tissue damage should consist of their therapeutic removal to a threshold value at which destructive inflammation is abated. Furthermore, the mechanism facilitating mediator and cytokine flow from the interstitial to the blood compartment during HVHF is unclear. The use of large replacement volumes for HVHF could promote and enhance lymphatic flow from the interstitium to the blood. Lymphatic flow can increase 20- to 40-fold when large fluid volumes (3 to 5 l/h) are infused [96]. Thus, the high fluid volume exchange in HVHF increases lymphatic flow, which directs mediators and cytokines to the blood compartment where they are subsequently removed, whereas HPHF, although able to remove larger amounts of mediators and cytokines from the blood, lacks the ability to influence processes outside the blood compartment. Of note is that increasing filter porosity might be a potentially effective method, because mediators have higher molecular weights than the cut-off points of conventional hemofilters [97]. In this respect, a recent study using a high cut-off (60 kilodaltons (kDa)) filter showed promising results [53]. Apparently, this is in contrast with HVHF and derived techniques, which address clearance of molecules below 45 kDa and with plasma filtration that targets molecules around 900 kDa. It is imperative to further develop methods that modulate the mediator spectrum between these two extremes [54]. Whether this technique may cause unwarranted loss of beneficial nutrients, hormones and drugs (e.g., antibiotics) is unclear. Large RCTs applying specifically designed high cut-off hemofilters are expected to explore this aspect of HPHF. Potential side-effects due to the undesired loss of vital substances could eventually be circumvented by the recently described hybrid techniques, such as CPFA and cascade hemofiltration (CCHF) [98]. Only target molecules within a relatively strict range of molecular weight are adsorbed and treated blood is returned to the patient. Except for one small study regarding safety [55], no large RCT has compared classic membranes with membranes exhibiting large pores, such as Septe*X*^® ^(Gambro™). A new generation of membranes has emerged that focuses on endotoxin adsorption (Toraymyxin^®^; Toray™ or Oxiris^®^; Gambro™) or specific immuno-adsorption (Prosorba^®^; Fresenius™). Preliminary results are promising [56], and future large RCTs are being prepared. As alluded in recent reviews, a more in-depth understanding of the mechanisms of blood purification removal is needed [99-101]. Finally, the type of vasopressor used for resuscitation may have an impact on apoptosis. During well-resuscitated septic shock after porcine peritonitis, low-dose arginine vasopressin is associated with less kidney damage by reducing both tubular apoptosis and systemic inflammation compared with noradrenaline [102].

### Conclusions and perspectives

New insights into the pathophysiology of septic AKI provide justification for future treatment studies. Exciting targets of intervention are the caspase cascade and intrarenal inflammatory pathways. During the past decade, hemofiltration has evolved steadily from a pure treatment for AKI to an important adjunctive therapy for sepsis and other inflammatory diseases (e.g., acute pancreatitis). More sophisticated technology along with a better understanding of immune-inflammatory pathways will more appropriately define hemofiltration doses and enhance efficacy of the devices. Actually, 35 ml/kg/h is considered to be the standard hemofiltration dose for treatment of septic AKI. However, in view of the high mortality rate in septic shock, a higher dose is required as salvage therapy in this condition. For non-septic-AKI, 25 ml/kg/h is the rule, but to achieve this goal 30 to 35 ml/kg/h must be prescribed. More data are eagerly awaited (especially the complete IVOIRE results) before a definite dose regimen can be recommended as routine ICU treatment of septic AKI. In recent years, many RRT modalities have been studied and developed for use in clinical sepsis. Manipulation of ultrafiltration dose, membrane porosity, mode of clearance, and combinations of techniques have all yielded promising findings. At present, conclusive evidence based on well designed, randomized, and controlled trials remains scarce, limiting implementation of these techniques in daily practice outside a study context. From the few studies so far, it can be concluded that optimalization of delivered dose in RRT has a proven positive effect. An ultrafiltration rate between 35 and 45 ml/kg/h, adjusted for predilution and down time, is recommended in sepsis. The results of further dose outcome studies using higher ultrafiltration rates will likely be the stepping stone to further improvements in daily clinical practice. In the near future, hybrid techniques most likely will expand the spectrum of RRT in sepsis. Recent information from substudies of the IVOIRE project suggests that starting treatment for septic AKI should be closer to rifle grade injury than failure. Large RCTs on various key topics are warranted to expand our knowledge and to decrease ultimately the unacceptably high mortality rate of ICU patients suffering from AKI.

## Competing interests

The authors declare that they have no competing interests.

## Authors' contributions

PMH, RJ, OJB, and HDS conceived and wrote the review. WB, JDR, EDW, and VC participated in literature search and selected appropriate articles. PMH and HDS participated in design, coordination, and writing. All authors read and approved the final manuscript.
